# Equine Multiple Congenital Ocular Anomalies and Silver Coat Colour Result from the Pleiotropic Effects of Mutant *PMEL*

**DOI:** 10.1371/journal.pone.0075639

**Published:** 2013-09-23

**Authors:** Lisa S. Andersson, Maria Wilbe, Agnese Viluma, Gus Cothran, Björn Ekesten, Susan Ewart, Gabriella Lindgren

**Affiliations:** 1 Department of Animal Breeding and Genetics, Swedish University of Agricultural Sciences, Uppsala, Sweden; 2 Department of Veterinary Integrative Biosciences, College of Veterinary Medicine and Biomedical Sciences, Texas A&M University, College Station, Texas, United States of America; 3 Department of Clinical Sciences, Swedish University of Agricultural Sciences, Uppsala, Sweden; 4 Department of Large Animal Clinical Sciences, College of Veterinary Medicine, Michigan State University, East Lansing, Michigan, United States of America; University of Sydney, Australia

## Abstract

Equine Multiple Congenital Ocular Anomalies (MCOA) syndrome is a heritable eye disorder mainly affecting silver colored horses. Clinically, the disease manifests in two distinct classes depending on the horse genotype. Horses homozygous for the mutant allele present with a wide range of ocular defects, such as iris stromal hypoplasia, abnormal pectinate ligaments, megaloglobus, iridociliary cysts and cataracts. The phenotype of heterozygous horses is less severe and predominantly includes iridociliary cysts, which occasionally extend into the temporal retina. In order to determine the genetic cause of MCOA syndrome we sequenced the entire previously characterized 208 kilobase region on chromosome 6 in ten individuals; five MCOA affected horses from three different breeds, one horse with the intermediate Cyst phenotype and four unaffected controls from two different breeds. This was performed using Illumina TruSeq technology with paired-end reads. Through the systematic exclusion of all polymorphisms barring two SNPs in *PMEL*, a missense mutation previously reported to be associated with the silver coat colour and a non-conserved intronic SNP, we establish that this gene is responsible for MCOA syndrome. Our finding, together with recent advances that show aberrant protein function due to the coding mutation, suggests that the missense mutation is causative and has pleiotrophic effect, causing both the horse silver coat color and MCOA syndrome.

## Introduction

**Figure 1:**
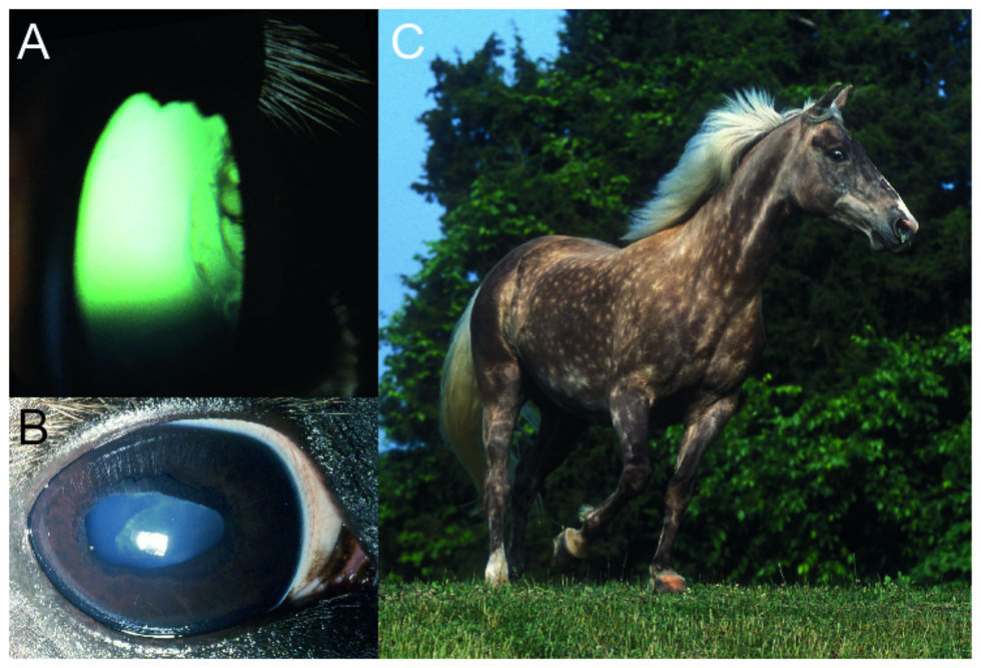
Clinical signs of MCOA syndrome and a Silver colored Rocky Mountain Horse. A) Single or multiloculated cysts originating from the ciliary body, iris and occasionally extending into the retina in the temporal quadrant of the eye is the hallmark of the Cyst phenotype and frequently observed in horses with the MCOA phenotype. However, in the MCOA phenotype, other intraocular anomalies, such as cataracts and mitotic pupils, may block the view of the temporal part of the posterior chamber and peripheral retina. B) The right eye of a Rocky Mountain Horse with ectropion uvea, dyscoria, cataract, and lens subluxation. The granula iridica is hypoplastic, the pupil is misshapen, and circumferential ectropion uvea is present. Nuclear cataract of the nuclearcortical junction is present. Vitreous is present in the anterior chamber between the iris and lens secondary to posterior ventral lens subluxation. C) A shiny white mane and tail, in conjunction with a slightly diluted body color with dapples, is typical of a genetically black Silver colored horse. This horse has also been diagnosed with MCOA. Reprinted from Andersson et al., BMC Genetics 2008, 9:88.

Equine Multiple Congenital Ocular Anomalies (MCOA) syndrome consists of different combinations of clinical symptoms that affect the anterior and posterior segment of the eye. While the syndrome was first described in the Rocky Mountain Horse, where it is highly prevalent, it is not limited to this breed. Additional horse breeds that have been diagnosed with MCOA syndrome include the Icelandic Horse, Shetland Pony, Exmoor Pony, American Miniature Horse, Belgian Draft and Morgan Horse, as well as the Kentucky Mountain Saddle Horse and Mountain Pleasure Horse, both of which are closely related to the Rocky Mountain Horse [1,2,3,4,5,6,7]. MCOA syndrome is inherited as an incompletely dominant trait. Horses with the MCOA phenotype are homozygous for the disease allele and have a wide range of eye anomalies (Figure 1a and b). These include, but are not restricted to, uveal cysts, cornea globosa, iris stromal hypoplasia, abnormal pectinate ligaments, cataracts and iris hypoplasia [1,8,9]. Horses that are heterozygous for the disease causing allele have the less severe Cyst phenotype which mainly consists of cysts that originate from the temporal ciliary body, iris, and/or extend into the temporal, peripheral retina [10,11]. The majority of MCOA syndrome horses also have the valued Silver coat color (Figure 1c). This coat phenotype is characterized by the dilution of black pigment in the hair and is most visible in the mane and tail. The Silver coat color is associated with a missense mutation in exon 11 of *premelanosome protein* (*PMEL*) [12,13]. It is not possible to see if a horse carries the *Silver* mutation on a chestnut background. However, these horses also display the disease phenotype. Selective breeding for the Silver coat color has simultaneously increased the frequency of MCOA syndrome in many breeds as both traits are closely linked on horse chromosome 6 [14].

Mutations in *PMEL* have previously been shown to regulate hypopigmented phenotypes in mice, chickens, zebrafish, dogs and cattle [15,16,17,18,19]. Only the zebrafish and dog have been reported to have concurrent ocular anomalies, although the clinical presentation in these species is quite different from that observed in the horse [18,20,21]. The zebrafish mutant, *fading vision*, is characterized by hypopigmented retinal pigment epithelium (RPE) and body melanocytes. Due to defects in RPE, the outer segments of photoreceptors are either strongly reduced in length or are completely absent. Interestingly, the retina of adults shows a partial recovery to normal morphology [18]. No abnormalities in the other structures of the eye were reported. *PMEL* mutant dogs are called “merles” and typically have blue eyes. The coats of heterozygous dogs are characterized by patches of diluted pigment intermingled with normal hairs, while the coat of homozygous dogs are predominantly white or very pale [19]. One study reported that 54.6% of double merle dogs had mild to severe deafness [22]. To a lesser extent homozygous merle dogs also have an elevated frequency of ocular anomalies including microphthalmia, microcoria, colobomas and increased ocular pressure [20,21]. Merle in the homozygous condition has also been reported to be sublethal due to negative pleiotropic effects on the skeleton, cardiac and reproductive systems [22]. However, not all double merles seem to be affected by theses pleiotropic effects. The reason for this may include modifying genotypes at other loci, strong linkage disequilibrium (LD) between the causative mutation and *PMEL* (which is positioned in a very gene dense region), or the length variation noted in the SINE element associated with the merle phenotype [22]. Unexpectedly, the SINE element has also been found in a few non-merle dogs [19].

The kinds of mutations identified in *PMEL* are both numerous and varied, and their phenotypic effects are quite diverse. It is so far not known if any of these mutations completely disrupt protein function. In order to study complete loss of function of PMEL, a mouse line with inactivated *Pmel* has recently been created [23]. This study showed that *Pmel* inactivated mice have a minor visible dilution of the coat color and that their melanosomes are spherical, instead of the rod shape present in wild-type animals. Interestingly, no ocular anomalies could be detected in these animals. The very subtle effects of completely disrupting PMEL suggests that *PMEL* mutant species with more pronounced phenotypic alterations, like the horse, carry dominant negative mutations. In yet another recent study of the biochemical properties of PMEL function, it was shown that mutations in or near the transmembrane domain of PMEL, like the *Silver* mutation in horse and *Dominant white* chicken, change the capacity of this region to self-associate [24]. These small in-frame mutations alter fibril formation into more compact structures and this in turn leads to severe pigment loss. Although the effect of the mutation causing the Dominant white phenotype in chicken was proved to be similar to that in the horse, ocular examination of these birds did not identify any ocular anomalies [25].

MCOA syndrome was previously mapped to a 208 kilo-base region on horse chromosome 6 (Andersson et al. 2011b). The region is gene dense and contains several genes that have been proposed to be involved in ocular development or disease. This study aimed at identifying the causative mutation of MCOA syndrome and in doing so, evaluate if *PMEL* is involved in this eye disease. Further, as the popularity of the Silver coat color is increasing across several horse breeds the results may also have important implications for informed breeding decisions. By next generation DNA sequencing we have managed to exclude all genes, except *PMEL*, as being causative for both the MCOA syndrome and the Silver coat color in horses.

## Results

### Generation of sequence data

In order to find the causative mutation for MCOA syndrome we deep sequenced five homozygous MCOA horses from three different breeds, one horse with the heterozygous Cyst phenotype and four unaffected controls (Table 1). In total we generated 36 Gb of sequence data (Study accession: http://www.ebi.ac.uk/ena/data/view/PRJEB4316, European Nucleotide Archive). For unknown reasons horse number 10, an unaffected Rocky Mountain Horse, generated almost five times more data (Table 1). On average, 93.4% of the reads from each horse could be mapped to the MCOA identity-by-decent (IBD) interval with an average coverage of 16,468 X. We did not analyze data from two smaller gaps of 100 and 640 base pairs (bp) respectively due to an inability to align reads to sequence gaps in the reference genome (0.3% of total sequence). These regions were subsequently sequenced with traditional Sanger technology, and analysis did not reveal any additional polymorphic sites. Except for these gaps, the coverage generated by Illumina sequencing was high at all positions, including those with high GC content, with a minimum depth of 25X at a few positions. All sequences within the MCOA interval were mined for SNPs, structural changes, and insertions and deletions (indels).

**Table 1:** Summary of data generated by Illumina sequencing for the MCOA syndrome identity-by-decent (IBD) region (ECA6: 73640494-73848154).

**ID**	**Phenotype**	**Breed**	**Total yield(Giga bases**)	**Mapped reads (%**)*	**Mapped reads to IBD-region (%**)	**Average coverage IBD- region (X**)
Horse 1	MCOA	American Miniature	2.4	95.4	93.1	10901.8
Horse 2	MCOA	Icelandic Horse	2.8	94.8	91.1	12624.0
Horse 3	MCOA	Icelandic Horse	2.8	97.8	96.0	13385.2
Horse 4	MCOA	Rocky Mountain Horse	3.0	96.6	94.5	13522.2
Horse 5	MCOA	Rocky Mountain Horse	2.4	94.6	92.4	11187.2
Horse 6	Cyst	American Miniature	2.8	94.1	90.7	12615.1
Horse 7	Unaffected	American Miniature	2.6	97.0	95.1	11493.6
Horse 8	Unaffected	American Miniature	2.4	94.9	92.4	11104.2
Horse 9	Unaffected	Rocky Mountain Horse	2.4	92.4	90.8	10489.8
Horse 10	Unaffected	Rocky Mountain Horse	12.4	97.9	96.5	57357.6

* Against the whole genome

### Polymorphism evaluation

In total we identified 609 SNPs, of which 23 were localized within conserved elements according to the 29 mammal dataset [26]. All ten re-sequenced horses were homozygous for the same allele at 60 of these SNPs, with the opposite allele only present within the reference genome. For indels we used quite a low quality filter even though this would give an increased number of false positives. Using this threshold we detected 129 possible indels. We manually inspected the reads of all indels positioned in conserved elements according to 29 mammal conservation scores and could confirm that eight out of 27 appeared real. We did not identify any structural rearrangements, large duplications or deletions.

### Association study

At either end of the sequenced IBD region there was a cluster of SNPs that could be used to clearly narrow the MCOA haplotype. Taken together, these excluded 14.4 kb, leaving a 193.3 kb IBD-region. At the 5’ end, the two Rocky Mountain Horses carried a different haplotype compared to the Icelandic and American Miniature Horses, while at the 3’ end, the Cyst-phenotype horse did not carry the MCOA associated haplotype.

In order to minimise the number of candidate mutations, optimum controls had been selected from a larger cohort of genotyped horses presented in an earlier study [14]. The criterion for inclusion as a control in the current study demanded a careful ocular examination and a haplotype similar to the disease haplotype. After we excluded all SNPs and indels that did not exactly match the correct inheritance patterns of the syndrome i.e horses with the MCOA-phenotype homozygous for one allele, Cyst-phenotype horse heterozygous and unaffected horses homozygous for the opposite allele, only two SNPs remained (Table 2). Both of these are positioned within the *PMEL* gene and have previously been associated with the Silver coat color [12,13]. One of these, g.73666064A>T is located in intron 9 and not within a conserved element. The hypothesis of a potential gain of function caused through the generation of a transcription factor motif was tested, however we could not find any supporting evidence that a known mammalian transcription factor binding site was created as a result of the SNP. The second SNP, g.73665304C>T, is positioned 760 bp downstream of the intronic SNP. This variant is not only conserved, but is also coding, positioned in the last exon of *PMEL*, exon 11. It introduces an amino acid change from an arginine to a cysteine (Arg625Cys) in the second position of the cytoplasmic region.

**Table 2:** Identified polymorphic sites which fitted the expected inheritance pattern and so qualified as candidate mutations for MCOA syndrome.

**Position**	**Reference allele**	**Mutant allele**	**Conserved**	**Coding**	**Predicted consequence**
chr6:73666064	A	T	no	no	none
chr6:73665304	C	T	yes	yes	Arg625Cys

In an effort to exclude the intronic SNP as causative we genotyped it in a random set of 936 horses. Of these, all Silver horses (n= 24) and some chestnut horses (n= 4) were heterozygous or homozygous for the T allele. Subsequent genotyping of the coding SNP revealed that they were either heterozygous or homozygous for the T allele in the coding SNP i.e. the alleles were in complete LD. None of the other 908 horses carried the T allele at the intronic SNP, demonstrating that this variant may be unique to the Silver haplotype.

## Discussion

This study establishes that *PMEL* is causative for MCOA syndrome. We were able to systematically exclude all other candidate genes from a previously identified 208 kb IBD-region using the results of deep sequencing, and in doing so clearly demonstrate the usefulness of this method for finding causative mutations, especially for monogenic disorders where there is a simple relationship between genotype and phenotype. A common outcome of many deep-sequencing projects is the large number of potential candidate mutations identified. In this study, we also show the benefits of well-selected controls. We genotyped control individuals for markers within and flanking each side of the established IBD-region and used this information to identify those that harboured either the ancestral haplotype or that were very close to the disease haplotype for sequencing. By doing so, we dramatically reduced the number of candidate mutations that required validation in additional animals. Further, by using long-range PCRs to build our libraries we were able to cover sequence that may have been missed with other technologies, such as genome gaps and long repeats, which are excluded during sequence capture. More than 90% of our reads mapped to the expected genetic interval with some loss likely due to poor alignment of repetitive sequences in the region.

The previously characterized MCOA syndrome region was 208 kb and contained 15 genes, of which several were potentially important for eye development. In this study we manage to exclude all polymorphic sites, barring two SNPs in *PMEL*, and propose that the causative mutation is a missense mutation in exon 11 which leads to an arginine to a cysteine (Arg625Cys) change in the second codon of the cytoplasmic region. PMEL is a pigment cell-specific integral membrane protein found in the melanosomes of cells synthesizing primarily the black and brown pigment, eumelanin [27,28]. Subsequent to cleavage, PMEL forms amyloid fibrillar sheets on which melanin is deposited [29]. These sheets form the structural foundation of eumelanosomes and define the organelles maturation stages [30]. The exact function of the fibrillar sheets is not completely understood but they are suggested to have a role in the processing of melanin intermediates, facilitating their polymerization, intracellular or intercellular transfer and/or detoxification [24,28,31]. The first cleavage that *PMEL* is subjected to breaks the protein into Mα and Mβ fragments. Paradoxically, the Mα fragment forms the actual fibrils important to melanin deposition while the horse missense mutation is within Mβ.

Fortunately, the molecular effects of the two dominant *PMEL* mutations, *Silver* in horse and *Dominant white* in chickens, have recently been studied in detail [24]. This has been accomplished by introducing mutations analogous to these in the context of human *PMEL* and subsequently evaluating protein function relative to the wild-type form. The horse mutation is positioned very close to the transmembrane domain (TMD) and whereas the wild-type TMD is not able to oligomerize, the mutant form gains this function and can readily self oligomerize. The horse mutation replaces the middle of three consecutive arginines with a cysteine. This new oligomerization ability of the TMD was therefore suggested to be a consequence of decreased electrostatic repulsion between the adjacent basic RRR motifs via the removal of a positive charge [24]. When examining the full-length PMEL, the TMD mutation did not alter protein trafficking or its ability to be processed into amyloidogenic Mα fragments. However, at the end of the cleavage process when a yet unidentified protease cleaves Mβ, there is a small difference in mutant versus wild-type PMEL processing. In wild-type PMEL, Mβ is digested into two different c-terminal fragments (CTF). In contrast, the mutant protein is predominantly digested into just one form, and this seems to be more stable. The consequence of this second finding remains to be evaluated, but may be less drastic than the gain of self oligomerization properties of the TMD [24].

Upon maturation of profibrils into elongated fibrillar sheets, human PMEL containing the analogous horse mutation shows marked differences to the wild-type. In mouse melanocyte cell lines expressing the latter, most stage III and IV melanosomes were densely pigmented. In cells expressing the mutant form, there was less space between the fibrils and the sheets appeared abnormally tightly packed. Exactly how the new TMD oligorization properties affects fibrillar sheet formation remains to be elucidated. Importantly though, and probably because of this arrangement, the organelles seemed to lack pigmentation. This finding suggests that either the fibrils were no longer capable of binding to melanins or melanin production was somehow inhibited. EM analysis revealed that cells expressing mutant PMEL were hypopigmented, harbored fewer pigmented melanosomes and were enriched in early stage melanosomes [24].

These data clearly demonstrate a significant functional consequence of the missense mutation and provide convincing evidence that the coding *PMEL* mutation is causative for both MCOA syndrome and the Silver phenotype in horses. The obvious further question involves how alterations in the properties of pigmented cells change the whole structure of the eye to a pathologic form. It is not yet known why *PMEL* mutations give ocular anomalies in some species such as dog, horse and zebrafish, but not in chicken, cow and mouse. This is an interesting topic for further research. Because the relatively mild phenotype in the null mice, all other known *PMEL* mutations are suggested to be dominant negatives [23].

The *silver* mutation in mice consists of a single nucleotide insertion that leads to a premature stop codon and a truncated protein, which is missing the last 25 C-terminal amino acids [16,32]. The mutation causes a dilution of eumelanin, which results in the premature graying of the hair due to the loss of follicular melanocytes [33]. In frame insertion/deletions in chicken *PMEL* are associated with the Dominant white, Dun and Smoky coat colors [17]. Dominant white chickens are completely white and have a nine base pair in frame insertion within the *PMEL* TMD. These chickens do not present with any ocular anomalies but the homozygotes appeared less pigmented in the outer layers of the choroid than their wild-type counterparts, while the pigment of the retina pigmented epithelium seemed to be unaltered [25]. Interestingly, a second mutation found in a flock of Dominant white chicken, causing the Smokey phenotype, dampens or prevents the accumulation of the pathogenic PMEL isoforms [24]. This mutation behaves like PMEL knockouts and has a much milder phenotype, again suggesting that it is better to have no PMEL at all than the TMD mutant forms. In cattle, dilution of coat color pigment is associated with a missense mutation as well as a three nucleotide, in frame deletion, both residing in the signal peptide of PMEL [15,34]. . The zebrafish mutant, *fading vision*, exhibits both defects in vision and hypopigmentation and has a point mutation in *PMEL*, which leads to a truncated protein [18]. The merle patterning of the domestic dog is characterized by patches of diluted pigment and is presumed to be caused by a retrotransposon insertion into intron 10 of *PMEL* [19]. Some homozygous merle dogs suffer from both auditory and ophthalmologic abnormalities, defects that are similar to those of the human auditory-pigmentation disorder, Waardenburg syndrome [20]. No mutations in human *PMEL* associated with pigmentation variation have yet been described, but they are likely to exist.

The link between coat color and eye development is not novel and several other known or suggested mutations for coat color have pleiotropic effects, as reviewed in Bellone [35]. In horses these include vision abnormalities (Congenital stationary night blindness in leopard spotted horses), deafness (frame overo patterning and sometimes splashed white patterning), defects in the nervous system (Lethal white foal syndrome in frame overo horses and Lavender foal syndrome) as well as in melanomas (in grey horses) [35,36]. The identification of the MCOA mutation is important not only for resolving the controversial function of *PMEL*, but it also has practical implications for horse breeders. Molecular testing of the Silver mutation can inform breeding decisions, which is especially meaningful for chestnut or related colors where it is impossible to know if individuals carry the mutation by coat phenotyping alone. Our advice to breeders is to avoid breeding Silver horses to each other. This will essentially eliminate the risk of breeding foals affected by the MCOA phenotype.

## Conclusion

The missense mutation g.73665304C>T in the cytoplasmic region of PMEL causes both MCOA syndrome and the Silver coat color. This conclusion is based on the fact that deep sequencing and subsequent analysis eliminated all other possible candidate genes from the IBD-region, and that functional data presented by others showed that the missense mutation causes PMEL function alterations and subsequent changed melanocyte structure.

## Materials and Methods

The study was approved by the Ethics Committee for Animal Experiments in Uppsala, Sweden as well as by the Institutional Animal Care and Use Committee at Michigan State University.

### Horses

Five horses with the MCOA phenotype (2 Icelandic Horses, 2 Rocky Mountain Horses and 1 American Miniature Horse), one horse with the Cyst phenotype (American Miniature Horse) and four unaffected controls (2 Rocky Mountain Horses and 2 American Miniature Horses) were selected for massively parallel Illumina sequencing. The horses were previously genotyped using a combination of SNP and microsatellite markers, both within and around the MCOA genetic interval, as part of another study [14]. A comparison between the MCOA haplotype and the haplotypes of each unaffected horse as well as for the horse with the Cyst phenotype is found in Table S1. The anterior and posterior segments of the eyes had also previously been examined by slit-lamp biomicroscopy and indirect ophthalmoscopy before and after induction of mydriasis with tropicamide eye drops [1,3]. The set of 941 random horses included in this study comprised American Miniature Horses (*n*= 8), Arabian Horses (*n*=19), Ardennes (*n= 21*), Cold Blooded Trotters (*n= 213*), Dole Horses (*n= 16*), French Trotters (n= 56) Gotland Ponies (*n= 29*), Icelandic Horses (*n= 185*), Kentucky Mountain Horses (*n= 23*), North Swedish Draft Horses (*n= 38*), Rocky Mountain Horses (*n= 17*), Shetland Ponies (*n= 30*), Standardbreds (*n= 174*), Swedish Warmbloods (n= 70) and Thoroughbreds (*n= 37*).

### PCR Amplification

Primer3plus [37] was used to design primers for a previously identified Identity-by-decent MCOA interval (EquCab2, Chr6: 73640494-73848154). The 208 kb region was amplified using 40 primer pairs (Table S2). Touch down PCR reactions (68°C-55°C, lowering -0.5°C/cycle) were carried in a total volume of 25 µl; 80 ng genomic DNA, 0.5 units KOD Hot Start DNA Polymerase (Novagen), 1.5 mM MgSO_4_, 1X KOD buffer, 0.20 mM dNTP, 5.0 µl Betain (Sigma B-0300), 0.28 mM forward primer, 0.28 mM reverse primer. Products were gel purified using Qiaquick Gel Extraction kit (QIAGEN) and amplicons from each horse were subsequently pooled.

### Library Preparation and Sequencing

Sequencing libraries were prepared from 1.5 µg of DNA according to the TruSeq DNA sample preparation guide #15005180 revA using reagents from the TruSeq DNA sample prep kit set A and set B v1 (Illumina). Briefly, DNA was fragmented using the Covaris S2 system (Covaris), overhangs were end-repaired followed by purification using AMPure XP beads (Beckman Coulter). An A-base was added to the blunt ends of the DNA fragments and adapters and indextags for sequencing ligated, followed by purification using AMPure XP beads. The DNA fragments and the library was size selected on a 2% agarose gel and the fraction containing a 500bp fraction containing the adapter ligated fragments was excised from the gel, purified using a QIAGEN gel extraction column (Qiagen) and amplified for 10 cycles of PCR, followed by purification using AMPure XP beads (Beckman Coulter). The quality of the libraries was evaluated using the Agilent Technologies 2100 Bioanalyzer and a DNA 1000-kit. The adapter-ligated fragments were quantified by qPCR using the Library quantification kit for Illumina (KAPA Biosystems) on a StepOnePlus instrument (Applied Biosystems/Life technologies) prior to 16 pM of the pooled libraries being subjected to cluster generation on the cBot instrument (Illumina Inc.) using TruSeq PE cluster generation kit v3. Paired-end sequencing was performed for 100 cycles in one lane using a HiSeq2000 instrument (Illumina Inc) and TruSeq SBS chemistry v3, according to the manufacturer’s protocols. Base calling was done on the instrument by RTA 1.12.4 and the resulting. bcl files were converted to Illumina qseq format with tools provided by OLB-1.9.0 (Illumina Inc.). To separate samples and PhiX control DNA sequenced in the same lane as the sample libraries, the qseq-files were demultiplexed, allowing for one mismatch. Both demultiplexing and mapping were done with CASAVA 1.7.0 (Illumina Inc.). Additional statistics on sequence quality were compiled from the base call files with an in-house script. Sequencing was performed by the SNP&SEQ Technology Platform in Uppsala, Sweden (www.sequencing.se).

### Data analysis

Standard methods for sequencing read alignments and variant detection were used. The Burrows-Wheeler Alignment tool (BWA) [38] was used for alignment to the horse genome (equCab2) [39]. Samtools software package [40] was used to analyse the aligned data. Data was first aligned, then converted to bam format, and subsequently sorted and indexed. We used BedTools [41] to calculate the depth and breadth of coverage for our selected target region with the coverageBed command. The mpileup command in Samtools [40] was used for SNP and indel calling (http://samtools.sourceforge.net/mpileup.shtml). The Buf command was used to keep the read depth. The Samtools program bcftools was used for SNP and indel calling and for output in the vcf format. Only qualities above 20 were included for further analyses based on a PHRED scale. Additional filtering with Varfilter was applied with minimum and maximum depth set at 50 vs. 100,000 to adjust for twice the average coverage. Indel calling generated some false positives (including real ones) that where analysed manually in Integrative Genomics Viewer (IGV, www.broadinstitute.org/software/igv/home) before exclusion. We also used Samtools pileup to extract the coverage for each single base for use in relative coverage calculations.

Further analyses of the data were performed with Seqscoring (http://www.seqscoring.org/) [42]. Detected SNPs and indels in vcf format were first scored by conservation across species. Constraint elements from 29 eutherian mammals [26] were used for comparison and scoring. The results from all samples were then merged into one file and displayed in the UCSC browser (http://genome.ucsc.edu/) to allow for comparison and interpretation in the case control association study. Finally, we performed a relative coverage assessment to identify larger insertions, copy number variations or deletions that differed between cases and controls. Samtools pileup files were used as input and output files were displayed in the UCSC browser (http://genome.ucsc.edu/) for interpretation.

### Evaluation of candidate mutations

Transcription factor binding site analysis was performed using CONSITE (http://asp.ii.uib.no:8090/cgi-bin/CONSITE/consite) [43]. The genotypes of the two *PMEL* mutations from the set of random horse breeds was assessed through Custom TaqMan SNP Genotyping assays (Carlsbad, USA). Primer and probe designs were obtained from ABI webpage (https://www5.appliedbiosystems.com/tools/cadt/). The following sequences were used to genotype the exon 11 SNP; forward primer: 5’-TCCATTGCTTACCAGTTTCCTTCTT-3’, reverse primer: 5’-GAGAGCTGAGCCCTGCTT-3’, reporter 1: 5’-CATAAGTCTGCGCCTGAT-3’, reporter 2: 5’-CATAAGTCTGCACCTGAT-3’. The following sequences was used to genotype the intronic SNP; forward primer: 5’-GCACAGCTTCAGTCAGTGTCT-3’, reverse primer: 5’-GGTAGGTACTTGGACAAGATCTAGGA-3’, reporter 1: 5’-CTGCCAGATAGCCTCT-3’, reporter 2: 5’-TGCCAGAAAGCCTCT-3’. The ABI PRISM 7900 HT sequence detection system for 384-well format was used for the analysis according to manufacturers’ instructions.

## Supporting Information

Table S1 **A comparison between the MCOA haplotype and the haplotype of unaffected horses as well as one horse with the cyst phenotype.**
(PDF)

Table S2 **Primers used to amplify the MCOA region.**
(XLSX)
