# Supplementary material for: Equine Multiple Congenital Ocular Anomalies and Silver Coat Colour Result from the Pleiotropic Effects of Mutant PMEL
Source: PLoS One. 2013 Sep 23;8(9):e75639. doi: 10.1371/journal.pone.0075639 (PMC3781063; doi:10.1371/journal.pone.0075639)
Supplement: Table S1 — A comparison between the MCOA haplotype and the haplotype of unaffected horses as well as one horse with the cyst phenotype. (PDF) [file pone.0075639.s001.pdf]

Table S1. **A comparison between the MCOA haplotype and the haplotype of unaffected horses as well as one horse with the cyst phenotype.**

ID	Phenotype	Breed	Colour	*MS1*	*MS14*	*PMEL17 (ex 11)*	*IKZF4 insA*	*MS3*	*TKY284*	*MS13*	*8453*	*8475*	*MS21*
Horse 1-5	MCOA	IS, RH, MINI	Multiple colours[A]	269 or 263	247	T	insA	231 or 233	177 or 175	222	A or G	T or A	253 or 261
Horse 6	Cyst	MINI	Chestnut Pinto	263/267	247/257	C/T		233/248	167/177	222/244	A/A	A/T	253/257
Horse 7	Unaffected	MINI	Blue Roan Pinto	263/267	247/247	C/C	insA/-	231/248	167/169	234/242	A/G	A/A	257/261
Horse 8	Unaffected	MINI	Chestnut	263/269	247/247	C/C	insA/-	233/256	169/169	254/254	A/G	A/T	255/261
Horse 9	Unaffected	RH	Black	267/269	247/257	C/C	insA/-	231/252	163/175	244/244			261/261
Horse 10	Unaffected	RH	Black	267/267	247/247	C/C	insA/-	231/248	161/175	222/246			261/263
Position[B]				73607795	73658168	73665305	73728912	73722621	73768488	73835084	73904952	73968182	74029118

Markers within the associated haplotype is marked in grey. IS= Icelandic Horse, RH= Rocky Mountain Horse, MINI= American Miniature Horse

[A]Silver Black, Chestnut and Palomino

[B]Genomic position on chromosome 6 according to equCab2
